# Sequential, Multifocal, and Delayed Avascular Necrosis of All Four Major Joints From Hips to Shoulders Following Corticosteroid Therapy

**DOI:** 10.7759/cureus.96317

**Published:** 2025-11-07

**Authors:** Okan Can Karadeniz, Muhammed Yusuf Afacan, Emre Özmen, Serdar Yuksel, Alican Baris

**Affiliations:** 1 Department of Orthopaedics and Traumatology, University of Health Sciences, Istanbul Physical Therapy and Rehabilitation Training and Research Hospital, Istanbul, TUR; 2 Department of Anatomy, Istanbul University-Cerrahpasa, Institute of Graduate Studies, Istanbul, TUR

**Keywords:** avascular necrosis humeral head, bilateral avascular necrosis, core-decompression, corticosteroid treatment, hip avascular necrosis, multifocal osteonecrosis, sequential changes, staged bilateral total hip arthroplasty

## Abstract

Osteonecrosis is a progressive disorder characterized by compromised bone perfusion leading to structural collapse and joint dysfunction. We describe a 59-year-old male patient who received a tapered regimen of oral methylprednisolone for facial palsy in 2012. Four months after treatment, he developed discomfort in the right hip, and imaging confirmed osteonecrosis of the right femoral head. Conservative management with nonsteroidal anti-inflammatory drugs (NSAIDs) and activity restriction was unsuccessful, leading to core decompression and later total hip arthroplasty (THA). Ten years later, he experienced pain in the contralateral hip, and imaging revealed osteonecrosis of the left femoral head, which was managed by THA. In 2025, the patient presented with pain and limited motion in the right shoulder. Magnetic resonance imaging demonstrated osteonecrotic lesions and marrow edema in the right humeral head, as well as a chronic focal lesion in the left humerus, despite the absence of symptoms. NSAID-based follow-up was selected for the right shoulder, while core decompression was planned for the left. This case illustrates delayed, multifocal, steroid-induced osteonecrosis progressively involving both hips and shoulders over more than a decade. This clinical scenario raises an important consideration: whether patients undergoing corticosteroid therapy should receive routine imaging of major joints to facilitate early detection of osteonecrosis and enable timely interventions, such as core decompression, that may delay or prevent progression to end-stage joint destruction requiring arthroplasty.

## Introduction

Osteonecrosis, also referred to as avascular necrosis (AVN), is a disabling disorder marked by the destruction of bone tissue as a result of compromised circulation [1,2]. The condition most commonly involves the hip joint, producing considerable pain, limitation of function, and if not managed appropriately, permanent structural damage [3]. However, in some cases, it may involve non-load-bearing joints such as the shoulder [1,3-5]. The incidence of humeral head osteonecrosis following corticosteroid exposure varies across studies, but is reported in up to 15-25% of cases after humeral fractures. Its pathophysiology is attributed to corticosteroid-induced microvascular compromise through mechanisms such as lipid deposition, fat embolism, and increased intraosseous pressure, ultimately leading to ischemia and bone cell death [1,2]. Although the most affected bone is the femur, the head of the humerus is the second most affected bone part in the body [3]. Osteonecrosis, also known as AVN or aseptic necrosis, is still an issue that has not yet been clarified. However, it has been disclosed that the usage of corticosteroids can cause osteonecrosis [6]. A significant number of patients take corticosteroids for numerous diseases including chronic obstructive pulmonary disease, inflammatory skin disease, rheumatoid disease, and in this particular case, peripheral nervous disease like facial palsy, also known as Bell’s palsy. Thus, they are at risk of developing AVN [7,8]. Different causes have been exemplified to provoke osteonecrosis such as alcohol abuse, trauma, Gaucher’s disease, Caisson disease, and diabetes [5]. Nevertheless, the most common and paramount cause is corticosteroids [4,5].

The purpose of reporting this rare case is to highlight several key considerations: first, that AVN may develop even a decade after corticosteroid therapy administered for facial paralysis; second, that AVN may involve not only the femoral head but also the humeral head; and third, that the disease can progress sequentially, affecting multiple major joints over time. By presenting this case, we also aim to draw attention to the important clinical question of whether patients receiving long-term corticosteroid treatment should undergo routine magnetic resonance imaging (MRI) surveillance for early detection of AVN, with the potential to intervene before advanced stages necessitate arthroplasty. We also sought to fill the gap in the literature regarding delayed, multifocal, and temporally progressive cases of AVN that may occur even at low doses, which are rarely reported.

## Case presentation

A 59-year-old male patient presented with symptoms of left-sided peripheral facial paralysis, including inability to close the left eyelid completely, drooping of the mouth corner, impaired forehead wrinkling, asymmetry of facial expressions, and difficulty with speech in 2012. For this reason, the patient was commenced on methylprednisolone. Steroid treatment consisted of oral methylprednisolone, at a dose of 1 mg/kg per day for four days, then reduced to 60 mg/day on the fifth and sixth days, 40 mg/day for the seventh and eighth and 20 mg/day until the 40th day, which makes 1450 mg prednisolone-equivalent in total. 

**Figure 1:**
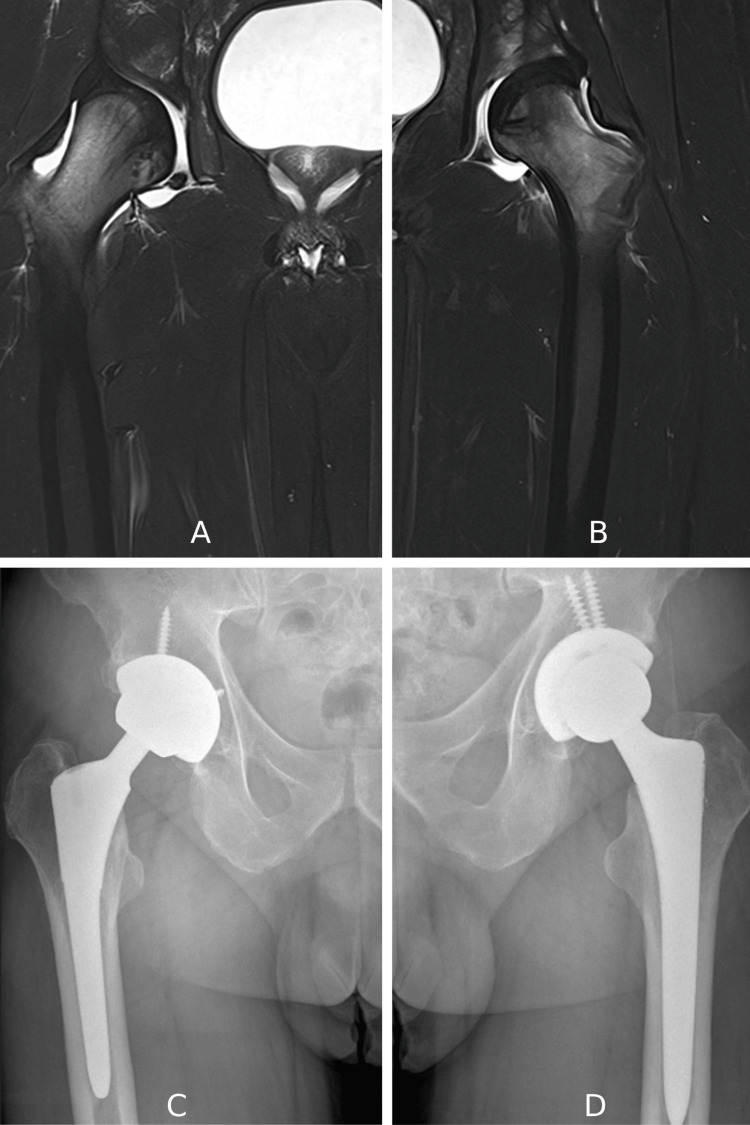
Magnetic resonance imaging (MRI) and radiographic findings of the bilateral hip osteonecrosis (A) Coronal MRI of the right hip showing a crescentic subchondral area of low signal intensity and underlying necrotic bone marrow changes. (B) Coronal MRI of the left hip depicting similar subchondral collapse and bone marrow necrosis. (C) Postoperative anteroposterior pelvic radiograph of the right hip demonstrating total hip arthroplasty. (D) Postoperative anteroposterior pelvic radiograph of the left hip showing total hip arthroplasty.

Approximately four months later, he presented with right hip pain, occurring after walking more than two blocks, while tying his shoes, and while climbing stairs. Initial evaluation included pelvic radiography and pelvic MRI, which revealed AVN of the right femoral head (Figure 1a).

The patient was managed with oral nonsteroidal anti-inflammatory drugs (NSAIDs) and advised non-weight bearing for four to six weeks, with gradual mobilization using bilateral crutches. Due to a prior history of tuberculosis, hyperbaric oxygen therapy was contraindicated. After two months without significant pain relief, the patient underwent core decompression of the affected femoral head. During the following year, his hip pain and restriction in the range of motion progressed, necessitating total hip arthroplasty (THA) of the right hip (Figure 1c). In 2022, the patient developed pain in the contralateral hip. Clinical findings were similar, such as pain during walking and climbing stairs. Clinical and radiological evaluations confirmed AVN of the left femoral head, for which THA was performed (Figures 1b, 1d).

**Figure 2:**
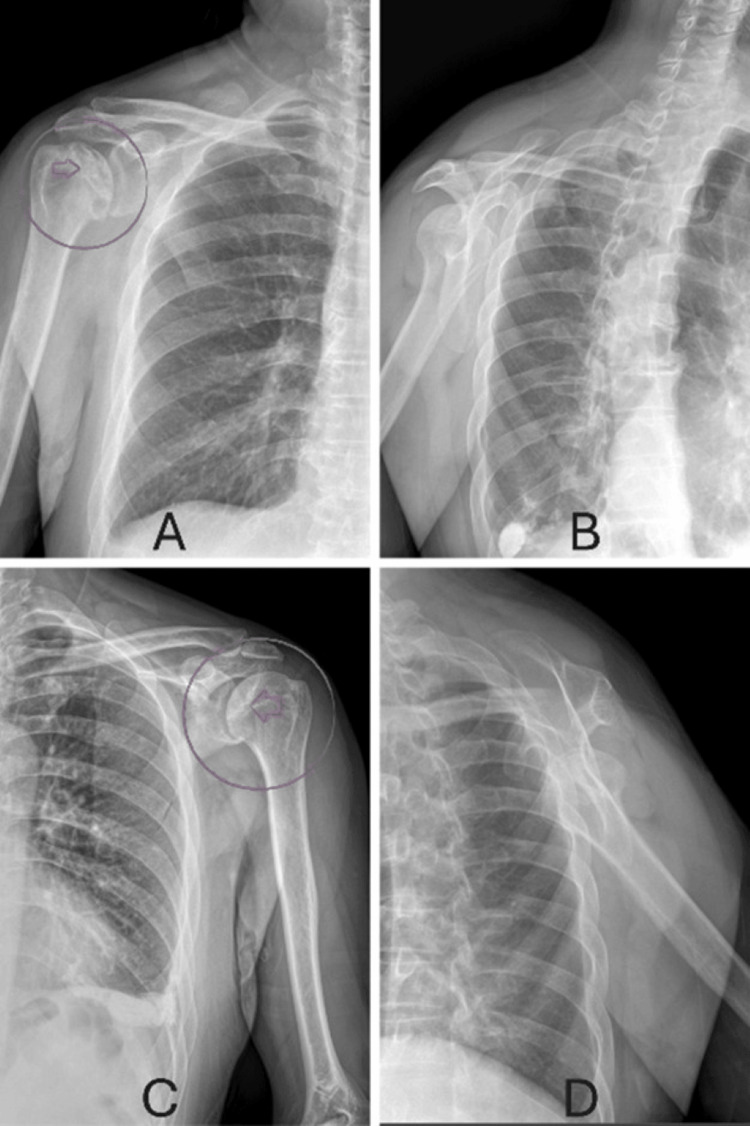
Radiographic findings of bilateral shoulder osteonecrosis (A) Anteroposterior radiograph of the right shoulder showing a crescentic subchondral radiolucent line (arrow, circled) (B) Scapular Y-view of the right shoulder (C) Anteroposterior radiograph of the left shoulder demonstrating a crescentic subchondral lesion (arrow, circled) (D) Scapular Y-view of the left shoulder

In 2025, the patient presented to our clinic with pain in the abduction and flexion of the right shoulder. Physical examination revealed pain and limitation on the Hawkins’ test, Neer’s test, and external rotation testing. The patient’s abduction was limited to 100 degrees and flexion was limited to 160 degrees. Initial X-ray showed changes in the humeral head, narrowing in the glenohumeral space (Figure 2a), relative humeral head migration (Figure 2b) and thus arthrosis on the right shoulder. 

Laboratory investigations at the time of diagnosis included complete blood count, liver and renal function tests, lipid profile, and coagulation parameters, all of which were within normal limits. Serum lipid evaluation showed triglyceride levels of 155 mg/dL (reference: <150 mg/dL) and total cholesterol of 210 mg/dL (reference: 90-200 mg/dL), suggesting mild dyslipidemia potentially associated with corticosteroid use. No evidence of thrombophilia or autoimmune disease was detected.

**Figure 3:**
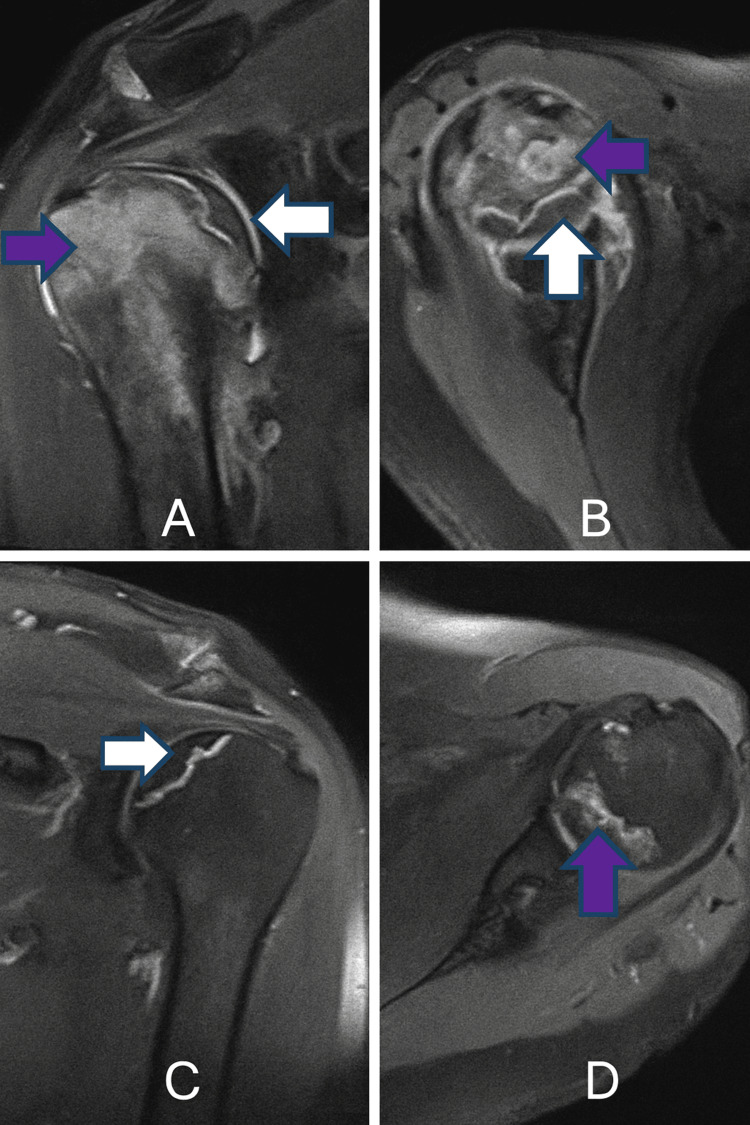
Magnetic resonance imaging (MRI) findings of bilateral shoulder osteonecrosis (A) Coronal MRI of the right shoulder showing a crescentic subchondral lesion (white arrow) and necrotic bone marrow (purple arrow). (B) Axial MRI of the right shoulder depicting similar crescentic subchondral changes (white arrow) and bone marrow necrosis (purple arrow). (C) Coronal MRI of the left shoulder illustrating a crescentic lesion beneath the articular surface (white arrow). (D) Axial MRI of the left shoulder demonstrating necrotic bone marrow within the humeral head (purple arrow).

Subsequently, radiographic and magnetic resonance imaging (MRI) evaluations were performed on both shoulders, despite the absence of any clinical symptoms in the left shoulder. The left shoulder X-ray demonstrated relative superior migration of the humeral head, comparable to that observed on the right side (Figure 2d). Both the X-ray and MRI of the left shoulder revealed a crescent sign, indicative of AVN (Figures 2c, 3c, 3d).

Additionally, an MRI of the right shoulder demonstrated minimal joint effusion, with inflammatory changes noted around the biceps tendon, within the subscapular bursa, and in the glenohumeral joint space. Areas of AVN, patchy bone marrow edema, and a crescentic subchondral lesion were also identified in the epiphyseal region of the right humeral head (Figures 3a, 3b).

This was classified as Stage 3 according to the ‘Cruess Classification of the Humeral Head Osteonecrosis’ [9]. In addition, a focal chronic AVN area and crescent sign observed in the medial epiphysis of the left humerus were also classified as Stage 1 according to the Cruess classification [9].

**Figure 4:**
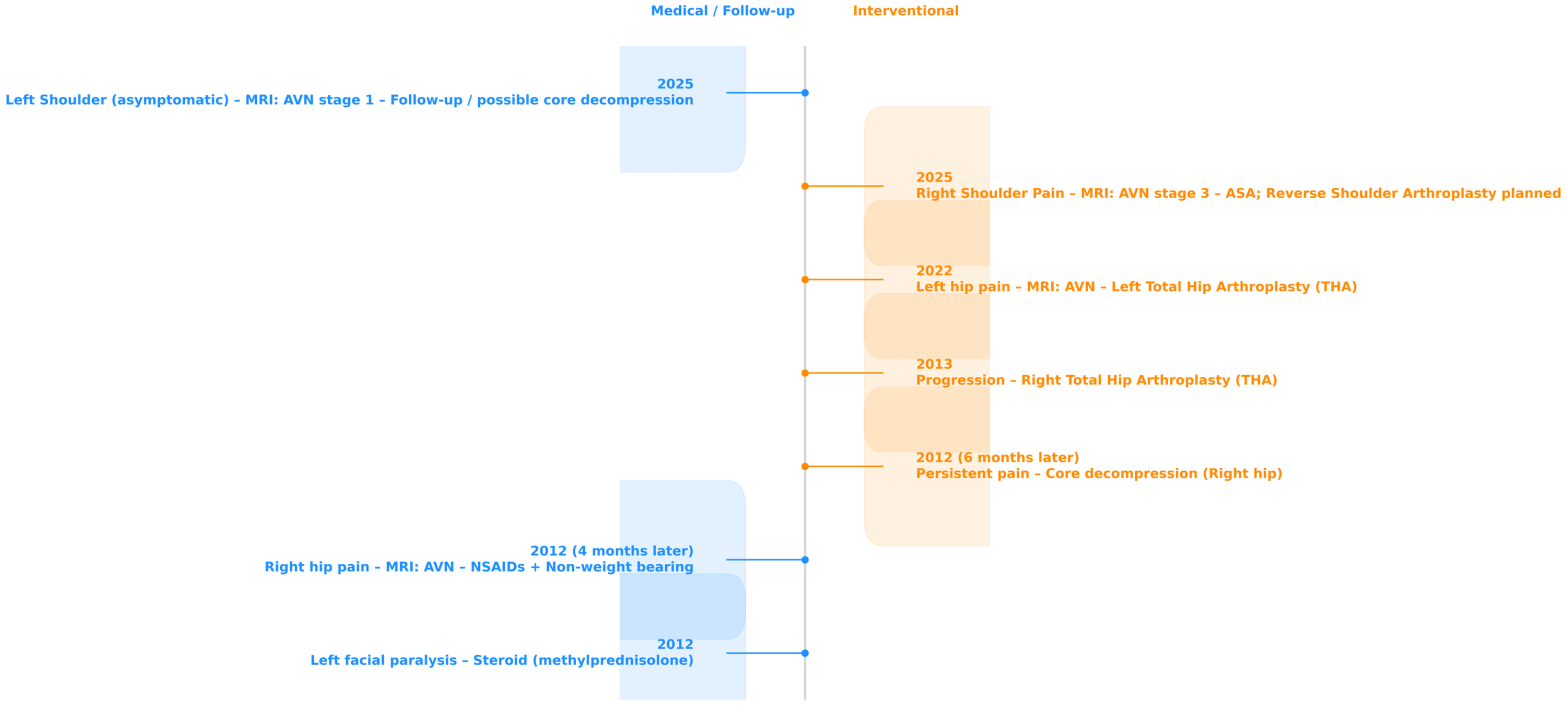
Timeline of steroid-induced multifocal avascular necrosis progression The diagram illustrates the sequential development of avascular necrosis (AVN) involving both femoral heads and both humeral heads over a 13-year period following corticosteroid therapy for facial palsy. Medical management such as non-steroidal anti-inflammatory drugs (NSAIDs) and follow-up events are indicated in blue, while surgical interventions (core decompression and arthroplasty) are marked in red. The patient initially developed right hip AVN four months after steroid treatment, progressing to core decompression and ultimately total hip arthroplasty (THA). The contralateral hip was affected a decade later, requiring THA. In 2025, right shoulder AVN (stage 3) led to follow-up with acetylsalicylic acid (ASA) and consideration of reverse shoulder arthroplasty, while asymptomatic left shoulder AVN (stage 1) was detected on MRI, with core decompression proposed as a joint-preserving option.

For the AVN in the patient’s right shoulder (Figures 3a, 3b), follow-up treatment with acetylsalicylic acid was considered appropriate, with reverse shoulder arthroplasty as an option if it failed, and core decompression was evaluated as a potentially beneficial option for the left shoulder, although the patient was asymptomatic. The patient remains under periodic clinical and radiologic follow-up, with stable findings and preserved function at the latest evaluation. The chronological sequence of AVN progression in all four major joints, along with the corresponding medical and surgical management, is summarized in Figure 4.

## Discussion

AVN is a condition resulting from the death of bone, cartilage, and connective tissues in the joint due to impaired perfusion [1,2]. Although precise pathophysiology remains incompletely elucidated, corticosteroid use is recognized as the leading non-traumatic predisposing factor [6]. A significant number of patients are taking corticosteroids for numerous diseases, including chronic obstructive pulmonary disease, inflammatory skin disease, rheumatoid disease and, in this particular case, peripheral nervous disease like facial palsy, also known as Bell’s Palsy. Thus, the literature extensively examines steroid-related AVN [4-6,10]. In our case, the patient received a total of 1450 mg prednisolone-equivalent over 40 days, with initial symptoms appearing approximately four months after therapy.

Although the femoral head remains the most commonly involved site, the humeral head is the second most frequently affected joint [3]. Multifocal involvement, particularly affecting both femoral and humeral heads, is uncommon [4,5]. In this patient, AVN progressively affected all four major joints over more than 10 years. Remarkably, the lesion in the left humerus was asymptomatic, highlighting the possibility of subclinical disease in individuals exposed to corticosteroids. Çavuş et al., in their case study, reported that osteonecrosis developed in four joints simultaneously (bilateral femoral heads and bilateral humeral heads) within two years following corticosteroid use [5]. The patient underwent core decompression for the hip joints, and clinical improvement was observed at the three-year follow-up [5]. In contrast, in our case, the onset of symptoms was distributed over 10 years, raising the question of whether patients receiving corticosteroid therapy should be monitored regularly for osteonecrosis in multiple joints.

This case underlines several clinical implications. Continuous surveillance of patients who receive corticosteroids is crucial, even after short-term or moderate-dose therapy. Multifocal AVN may remain clinically silent in certain joints, raising the question of whether MRI evaluation of all major joints should be considered in high-risk individuals. Moreover, the mechanisms leading to simultaneous involvement of multiple joints are not fully understood and may include genetic susceptibility, local variations in bone perfusion, or systemic factors affecting bone metabolism.

In patients diagnosed with early-stage AVN, conservative approaches such as restricted weight-bearing and activity modification may be feasible, while NSAIDs and bisphosphonates can also provide therapeutic benefits [1,2]. Additionally, physical therapy and rehabilitation remain potential options in the later course of management [1,2]. In cases where these interventions fail to achieve satisfactory outcomes, surgical procedures such as core decompression, vascularized bone grafting, or THA may become necessary [2,3]. Therefore, research focused on multifocal AVN development following low-dose steroid use, as well as efforts toward early diagnosis, could allow patients to receive less invasive treatment options while simultaneously offer cost-effectiveness.

Compared with previously published cases, the present report differs in both chronology and extent of involvement. Çavuş et al. described synchronous four-joint involvement within two years of steroid exposure, whereas our patient exhibited a delayed, sequential progression spanning over a decade. Heimann and Freiberger first reported steroid-induced multifocal AVN in 1960, but few subsequent studies have documented such long latency after a single moderate-dose regimen. Moreover, while most cases involve hips or knees, our case uniquely demonstrates delayed humeral head lesions after prior bilateral hip arthroplasties, underscoring the need for lifelong surveillance [4,5].

Finally, our case draws attention to a gap in the literature. The rarity of steroid-induced AVN involving all four major joints underscores the need for further investigation to determine whether close monitoring of large joints in corticosteroid users, regardless of therapy duration, is beneficial. Key questions remain regarding whether asymptomatic joints should be routinely imaged, how to stratify risk among corticosteroid recipients, and which interventions may prevent progression in subclinical lesions.

## Conclusions

This case demonstrates a rare presentation of multifocal AVN involving both femoral and humeral heads, occurring sequentially over a span of more than 10 years following a moderate, short-term corticosteroid regimen prescribed for peripheral facial paralysis. The patient’s disease course shows that even limited corticosteroid exposure may result in delayed, multifocal joint involvement. Despite the absence of shoulder symptoms, MRI imaging confirmed humeral head lesions consistent with AVN, highlighting the potential for subclinical progression. The long interval between steroid exposure and subsequent joint manifestations highlights the necessity of individualized, long-term follow-up in similar patients. Early radiological evaluation in asymptomatic joints, as in this case, may facilitate timely recognition and intervention before irreversible joint destruction occurs.
